# Structural insights into binding of polyglutamylated tetrahydrofolate by serine hydroxymethyltransferase 8 from soybean

**DOI:** 10.3389/fpls.2024.1451839

**Published:** 2024-08-19

**Authors:** Luckio F. Owuocha, Melissa G. Mitchum, Lesa J. Beamer

**Affiliations:** ^1^ Department of Biochemistry, University of Missouri, Columbia, MO, United States; ^2^ Department of Plant Pathology and Institute of Plant Breeding, Genetics, and Genomics, University of Georgia, Athens, GA, United States

**Keywords:** enzyme, enzyme structure, polyglutamylation, structural biology, structure-function, X-ray crystallography, crystal structure

## Abstract

Tetrahydrofolate and its derivatives participate in one-carbon transfer reactions in all organisms. The cellular form of tetrahydrofolate (THF) is modified by multiple glutamate residues and polyglutamylation plays a key role in organellar and cellular folate homeostasis. In addition, polyglutamylation of THF is known to increase the binding affinity to enzymes in the folate cycle, many of which can utilize polyglutamylated THF as a substrate. Here, we use X-ray crystallography to provide a high-resolution view of interactions between the enzyme serine hydroxymethyltransferase (SHMT), which provides one carbon precursors for the folate cycle, and a polyglutamylated form of THF. Our 1.7 Å crystal structure of soybean SHMT8 in complex with diglutamylated 5-formyl-THF reveals, for the first time, a structural rearrangement of a loop at the entrance to the folate binding site accompanied by the formation of novel specific interactions between the enzyme and the diglutamyl tail of the ligand. Biochemical assays show that additional glutamate moieties on the folate ligand increase both enzyme stability and binding affinity. Together these studies provide new information on SHMT structure and function and inform the design of anti-folate agents.

## Introduction

**Figure 1:**
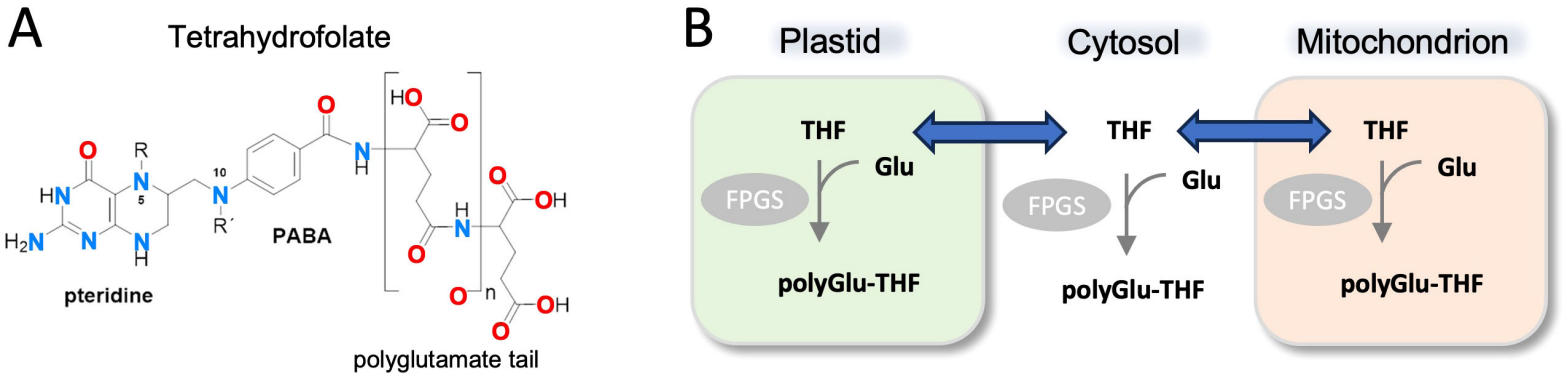
Structure of THF and overview of THF polyglutaymylation. **(A)** Molecular structure of THF including the variable poly(γ-glutamyl) modification and its γ-peptide linkage through the sidechain. The N5 and N10 nitrogen atoms are numbered for reference. PABA: p-aminobenzoic acid. **(B)** Simplified schematic showing transport of THF between cellular compartments such as the cytosol, mitochondria, and plastid (in plants). Modification of THF with poly(γ-glutamyl) groups occurs within cellular compartments and is catalyzed by the enzyme folylpolyglutamate synthase (FPGS). Adapted from (Garza-Aguilar et al., 2020).

Folates act as donors and acceptors in one-carbon metabolism and play essential metabolic roles in all organisms. In most cells, tetrahydrofolate is modified by a variable number of glutamate residues linked as amides through the γ-carboxyl group, a covalent modification known as polyglutamylation. Polyglutamylation of THF has known roles in cellular homeostasis and regulation of folate metabolism (McGuire and Bertino, 1981; Schirch and Strong, 1989). As polyglutamylated THF (polyGlu-THF) does not cross cellular membranes, this modification serves to maintain distinct pools of folate in different cellular compartments and also blocks export of THF from cells (**Figure 1**). The number of glutamates linked to folate is variable depending on organism, cell type, and sub-cellular location, ranging from one to more than 15 (Garza-Aguilar et al., 2020), but is often around five to six. Polyglutamylation of folate affects diverse processes, such as maintenance of the mitochondrial genome (Cherest et al., 2000), chromatin silencing (Zhou et al., 2013) and fruit development and ripening (Garza-Aguilar et al., 2020). Folate polyglutamylation has been linked to substrate channeling for some enzymes in the folate cycle (Schirch and Strong, 1989), and is a consideration in the design of anti-folate agents (Ruszkowski et al., 2019; Katinas et al., 2024).

The enzyme serine hydroxymethyltransferase (SHMT) plays a key role in folate-mediated one carbon metabolism, providing precursors for multiple metabolic pathways. SHMT catalyzes the reversible conversion of L-serine and THF into glycine and 5,10-methylene THF. SHMT is highly conserved in evolution, utilizes pyridoxal 5-phosphate (PLP) as a co-factor and can accept polyGlu-THF as a substrate (Matthews et al., 1982; Schirch and Strong, 1989; Strong et al., 1989). Prior studies of SHMT have indicated that polyGlu-THF binds to the enzyme with higher affinity than the monoglutamylated form (Schirch and Ropp, 1967; Matthews et al., 1982; Rebeille et al., 1994; Huang et al., 1998; Wei et al., 2013). However, despite relevance to enzyme function and folate metabolism, structural interactions between SHMT and polyGlu-THF are poorly characterized (Fu et al., 2003). These are of particular importance for inhibitor design, as SHMT is a target for antibacterial, antimalarial, anti-cancer, and herbicide agents (Schwertz et al., 2017, 2018; Batool et al., 2020; Cuthbertson et al., 2021; Zarou et al., 2021; Makino et al., 2022; Jin et al., 2023; Situ et al., 2023; Li et al., 2024).

Here, we have used X-ray crystallography and biochemical studies to gain insight into the interactions of polyGlu-THF with SHMT8, a cytosolic enzyme isoform from *Glycine max* (soybean). We present a 1.7 Å resolution structure of soybean SHMT8 in ternary complex with PLP-glycine (PLP-Gly) and the diglutamylated form of 5-formyl-THF (diGlu-FTHF). Through comparisons with the known complex with (monoglutamylated) FTHF (PDB ID: 6UXJ) (Korasick et al., 2020), we identify interactions specific to the SHMT8 – diGlu-FTHF complex, including a conformational change in a key loop at the entrance of the folate binding site. The novel loop conformer enables multiple interactions with atoms in the two glutamate moieties that are not present in the complex with FTHF. Biochemical studies of enzyme thermal stability and folate binding affinity support observations from the crystal structure. Implications for the design of anti-folate inhibitors for SHMT are discussed.

## Material and methods

### Protein purification and crystallization

Soybean SHMT8 (cv. Essex) was expressed in *E. coli* and purified as previously described (Korasick et al., 2020). For crystallization experiments, SHMT8 samples were prepared at 10 mg mL^-1^ in 25 mM HEPES, pH 7.5, 50 mM NaCl, and 0.5 mM Tris(carboxyethyl)phosphine (Hampton Research) and supplemented with 0.25 mM PLP (Sigma Aldrich). To obtain a ternary complex with PLP-Gly and diGlu- or tetraGlu-FTHF (Schircks Laboratories, Bauma, Switzerland), stock solutions of ligands were prepared in the same buffer as the protein. Glycine at 20 mM and diGlu-FTHF or tetraGlu-FTHF at 6 mM or 8 mM final concentration, respectively, were added to the protein. Crystallization screens were performed at 19°C using the hanging-drop vapor-diffusion method with a 1:1 ratio of the protein-ligand solution to the reservoir solution (total volume 4 μl). Initial crystals with diGlu-FTHF were obtained from condition 92 of the Index screen (Hampton Research) containing 0.1 M magnesium formate dihydrate and 15% (w/v) PEG 3350. Microseeding was done to increase crystal size, using a 1:3:4 ratio of micro seeds: protein: reservoir solution (total volume 4 μl). Data collection quality crystals with glycine and diGlu-FTHF were obtained in 0.1 M magnesium formate dihydrate and 13 - 17% (w/v) PEG 3350 within 48 hours. Data collection quality crystals were not obtained with tetraGlu-FTHF. Crystals were cryoprotected through the addition of 100% (v/v) ethylene glycol to the crystallization drop to a final concentration of 30% (v/v) ethylene glycol and flash-cooled in liquid nitrogen.

### X-ray diffraction data collection, phasing, and refinement

X-ray diffraction data were collected at the Advanced Light Source at beamline 4.2.2 using a Pilatus 3 6M detector in shutterless mode. The data set consists of 720 images spanning 180°. The data were integrated and scaled with XDS (Kabsch, 2010), and intensities merged and converted to amplitudes with Aimless (Evans and Murshudov, 2013). Initial phases for the diGlu-FTHF complex with SHMT8 were determined through Fourier synthesis and refinement was begun using the structure of SHMT8 bound to FTHF (PDB ID: 6UXJ; 100% sequence identity) (Korasick et al., 2020) as the template, after removal of ligands and water molecules. The model was improved using the PHENIX software package (Adams et al., 2010) via iterative rounds of refinement with phenix.refine (Afonine et al., 2012) and manual building in COOT (Emsley and Cowtan, 2004). The structures were validated using MolProbity (Chen et al., 2010) and the wwPDB validation service (Gore et al., 2017). Modeling of ligands was validated with polder omit maps (Liebschner et al., 2017). Data collection and refinement statistics may be found in **Table 1**.

**Table 1:** X-ray diffraction data collection and refinement statistics.

Space group	*P*2_1_
Unit cell parameters (Å, °)	a = 86.208, b = 89.02, c = 146.19 β = 90.536
Wavelength (Å)	1.07216
Resolution (Å)	43.1 - 1.69 (1.75 - 1.69)
Total reflections	822007 (27295)
Unique reflections	238721 (10106)
Multiplicity	3.4 (2.7)
Completeness (%)	96.72 (87.07)
Mean I/σ	8.6 (0.6)
Wilson B-factor (Å^2^)	23.49
*R*_merge_(*I*)	0.066 (1.057)
*R*_meas_(*I*)	0.091 (1.442)
*R*_pim_(*I*)	0.048 (0.873)
CC_1/2_	0.997 (0.314)
*R*_cryst_	0.1881 (0.2976)
*R*_free_*	0.2160 (0.3231)
Number of non-hydrogen atoms	16518
macromolecules	14564
ligands	476
solvent	1478
Protein residues	1888
rmsd bonds (Å)	0.007
rmsd angles (°)	0.97
Ramachandran favored (%)	97.45
Ramachandran allowed (%)	2.55
Ramachandran outliers (%)	0.00
Clashscore	4.35
Average B-factor (Å^2^)	29.40
macromolecules	28.54
ligands	29.78
solvent	37.74
PDB code	8TQF

Statistics for the highest-resolution shell are shown in parentheses. *5% test set used for R_free_ calculations.

### Folate binding assay

The binding affinity of polyGlu-FTHF compounds to SHMT8 was determined as previously reported (Korasick et al., 2020). Briefly, single enzyme solutions at 20 µM were prepared with diGlu-, tetraGlu-, or hexaGlu-FTHF (Schircks Laboratories) at concentrations ranging from 10 – 80 µM. (Assays with Forrest SHMT8 were only done with tetraGlu-FTHF). The glycine concentration was held constant at 5 mM. Protein and ligand solutions were mixed in a ratio of 1:1 in a 96-well plate. The plate was analyzed by an absorbance scan from 450–550 nm in a BioTek Epoch II plate reader following the formation of a peak at 500 nm, consistent with the formation of the quinonoid intermediate and representative of the total ternary complex in the enzyme solution (Strong et al., 1989; Cai et al., 1995). The absorbance at 500 nm was baseline subtracted from the absorbance at 540 nm. Data were analyzed using Origin 2023 by plotting absorbance versus polyGlu-FTHF concentration. Data for FTHF binding were fit to the Michaelis–Menten equation, using *K*_d_ = *K*_m_. Due to apparent non-Michaelis-Menten behavior, data for binding of the diGlu-, tetraGlu-, and hexaGlu-FTHF were fit to the substrate inhibition equation in Origin 2023. Errors represent the uncertainties of the parameters generated by the fitting program for the respective equations.

### Thermal shift assays

SHMT8 samples were diluted to 1.0 mg/mL (20 µM) in 50 mM HEPES, pH 7.4, 0.5 mM TCEP, and 50 mM NaCl in the presence and absence of various ligands. A total of six protein samples were prepared: protein in the absence of ligands; protein with glycine; and protein with glycine and FTHF, diGlu-, tetraGlu-, or hexaGlu-FTHF. The final concentrations for protein, glycine, and various forms of FTHF were 10 µM, 12.5 µM, and 1.25 µM, respectively. The samples were incubated with SYPRO Orange dye from the Applied Biosystems Protein Thermal Shift kit per the manufacturer’s recommendation for 1 hour at 4°C. A Quant Studio 3 Real-Time PCR System was employed to raise the temperature from 20°C to 99°C in intervals of 0.1°C, with a 10-second hold between each step. Experiments were conducted in triplicate, averaged, and fluorescence values normalized as described by (Andreotti et al., 2015). Curves were plotted using Origin 2023. T_0.5_ was determined as the midpoint of the normalized fluorescence response by fitting to the Boltzmann equation; errors reported are standard error of mean (SEM), where SEM = s/√n , *s* is standard deviation and *n* is number of observations.

### Software used

Molecular figures were prepared using PyMol (DeLano, 2002). Surface electrostatic potential was calculated at pH 7.0 using the PDB2PQR and APBS servers at: https://server.poissonboltzmann.org/ using default parameters (Jurrus et al., 2018) and displayed using PyMol (DeLano, 2002). Animation was created using the morph tool of ChimeraX (Meng et al., 2023).

## Results

### Overview of SHMT8 structure and its THF-binding site

**Figure 2:**
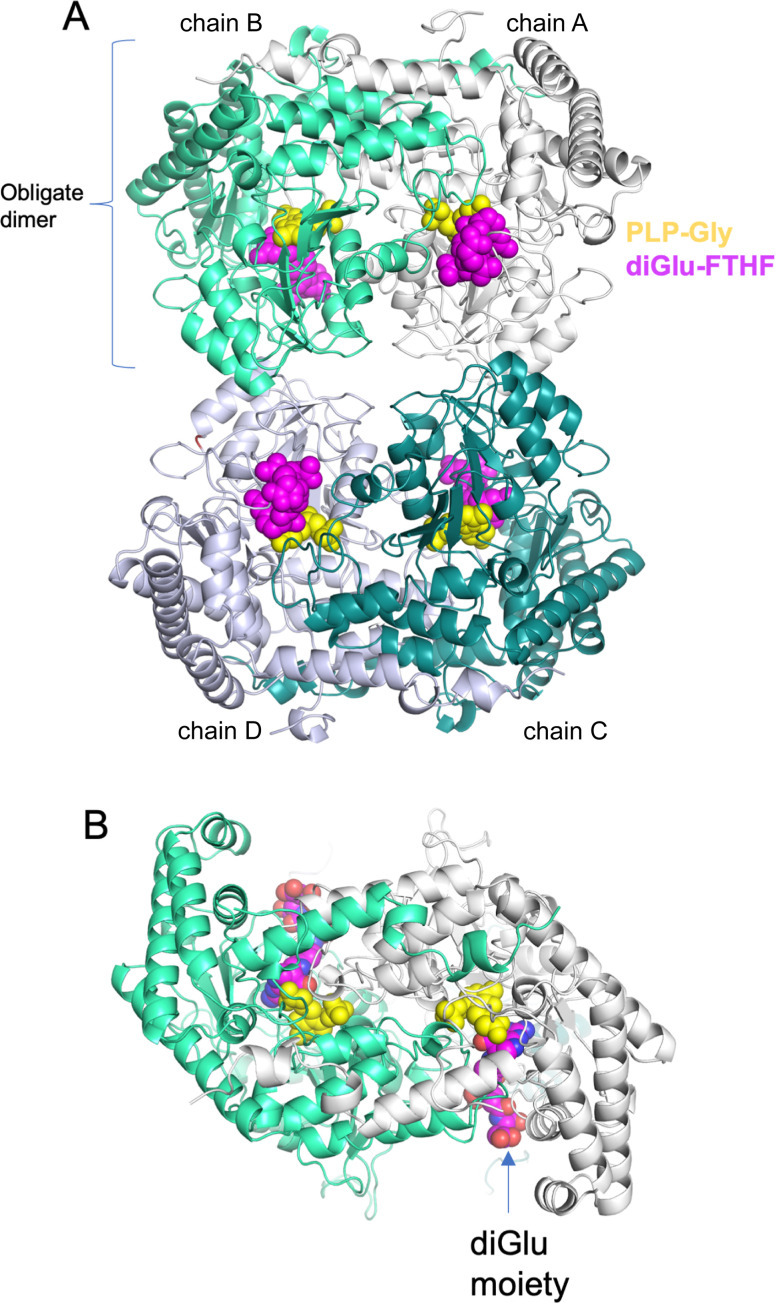
Overview of the crystal structure of soybean SHMT8 in ternary complex with PLP-Gly and diGlu-FTHF. **(A)** A cartoon diagram with the four chains of the tetramer in different colors: chain A - white; chain B – mint green; chain C - dark cyan; chain D - gray. Ligands are shown in space filling models: PLP-Gly (yellow) and diGlu-FTHF (magenta). **(B)** View of the obligate dimer (90° rotation from panel **A**) showing how the diglutamyl moiety is found at the surface of the protein.

Previous structural studies of soybean SHMT8 have provided detailed snapshots of the enzyme, representative of different stages of its multi-step reaction: the resting state of the enzyme with PLP; the enzyme bound to the PLP-Gly intermediate; and a ternary complex of the enzyme with PLP-Gly and the substrate analog FTHF (Korasick et al., 2020). These structures showed that, similar to SHMT from other eukaryotes, soybean SHMT8 adopts a tetrameric assembly, verified in solution by small-angle X-ray scattering and analytical ultracentrifugation (Korasick et al., 2020). The tetramer is composed of two obligate dimers (**Figure 2**). The two protomers within an obligate dimer are tightly intertwined, and residues from both polypeptide chains contribute to each active site (**Figure 2B**). In the ternary complex, PLP-Gly is in a deeply buried pocket at the interface of the two polypeptide chains. The FTHF substrate analog binds with its pteridine ring adjacent to PLP, as necessary for catalysis, while the para-amino benzoic acid (PABA) lies in a hydrophobic channel. The glutamate moiety of FTHF is positioned at the entrance of the THF binding site and is largely exposed to solvent. For a more detailed discussion of the SHMT8 catalytic site, see (Korasick et al., 2020).

**Figure 3:**
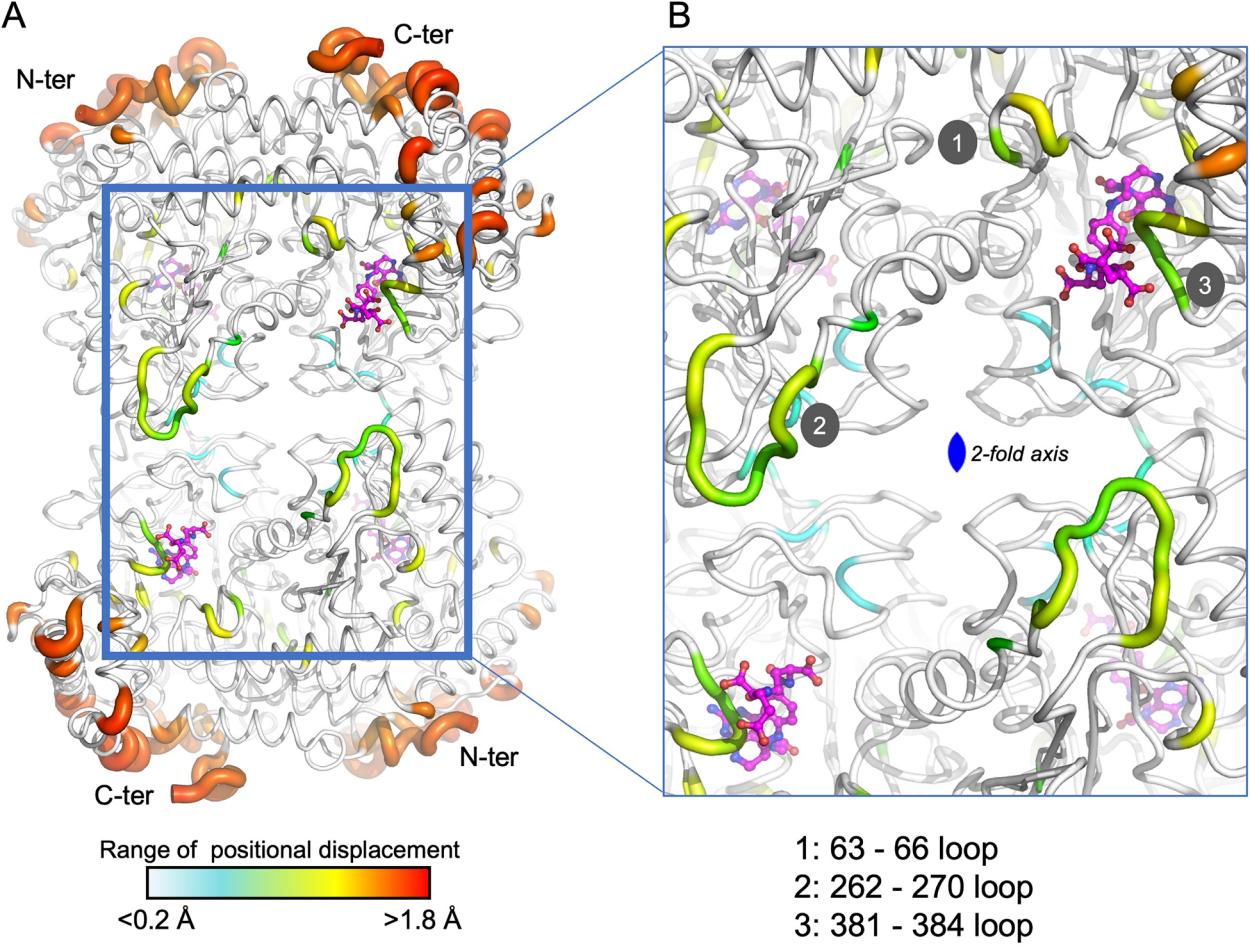
A tube diagram of the SHMT8 tetramer in complex with PLP-glycine and diGlu-FTHF. The diameter of the tube is proportional to the difference in atomic positions of the C_α_ atoms in a superposition of the FTHF and diGlu-FTHF complexes and is colored according to the largest (red) to smallest (blue) differences. **(A)** The entire tetramer showing the diGlu-FTHF ligands as ball-and-stick models. The flexible N- and C-termini are labeled for each protomer. Color bar indicates range for the positional displacements. **(B)** A closeup of regions near the folate-binding site highlighting three loops with intermediate structural changes (yellow/green) relative to the FTHF complex. Loops 1-3 are labeled; see text for details.

In this study, we have determined the crystal structure of soybean SHMT8 in complex with diGlu-FTHF and PLP-Gly at 1.7 Å resolution (**Table 1**). The diGlu-FTHF complex (PDB ID: 8TQF) crystallizes isomorphously with the previously determined FTHF ternary complex (PDB ID: 6UXJ) in space group *P*2_1_ with a single tetramer in the asymmetric unit. Both the PLP-Gly and diGlu-FTHF ligands are bound in all four active sites of the tetramer with overall occupancies of 1.0, and have similar binding poses to the ternary complex with FTHF. A superposition of the polypeptide chains in FTHF and diGlu-FTHF complexes yields a C_α_ root mean square deviation (rmsd) of 0.48 Å over 1892 residues, highlighting their overall structural similarity. However, several minor conformational changes occur relative to the FTHF structure (**Figure 3**). Aside from residues in the flexible N- and C-termini of the protomers (red/orange in **Figure 3A**), these changes are generally clustered in/around the entrance of the folate binding site (see loops 1, 3 in green/yellow in **Figure 3B**). A small shift is also seen in the nearby extended loop comprising residues 262-270 (loop 2). These changes are similar in all four polypeptide chains of the tetramer, maintaining the symmetry observed in the ternary complex with FTHF (Korasick et al., 2020).

### Conformational change of the THF-binding loop and Leu383

Of the three loops noted above, one is of particular interest in SHMT function: loop 3, which spans residues 381-384 and has also been called the THF-binding loop (residues 377-385) (Korasick et al., 2020). This loop was previously noted for conformational flexibility/changes in SHMT8. In particular, it adopts an ordered, closed conformation in the SHMT8 ternary complex with PLP-Gly and FTHF, when compared to structures without bound FTHF where it is largely disordered (Korasick et al., 2020, 2024). Moreover, two amino acid polymorphisms of SHMT8 that flank the THF-binding site and reduce its flexibility were shown to interfere with folate binding (Korasick et al., 2020), demonstrating its functional importance.

**Figure 4:**
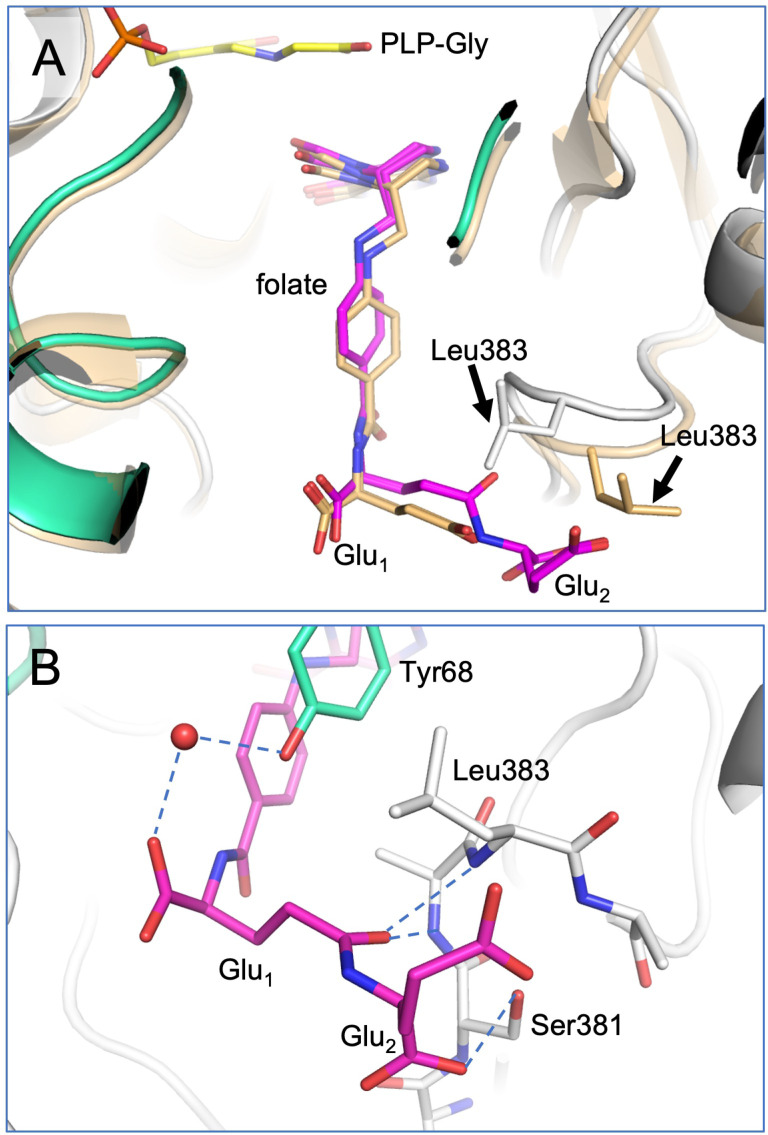
Closeup of diGlu-FTHF in its binding site with SHMT8. **(A)** A superposition of the FTHF and diGlu-FTHF complexes in the vicinity of the folate binding site. The FTHF ligand and protein (PDB ID: 6UXJ) are in tan. The diGlu-FTHF ligand is in magenta with chains A/B of the protein shown in white/mint green. Residues 381-384 change position in the diGlu-FTHF complex moving closer to the para-aminobenzoic acid ring of the folate. The position of the Leu383 sidechain in both complexes is highlighted by arrows. **(B)** Novel hydrogen bonds between the Glu_1_ and Glu_2_ moieties and enzyme. Note position of Leu383 (chain A) sidechain where it approaches the ring of Tyr68 (chain B).

In the SHMT8 complex with diGlu-FTHF, we identify an additional conformational change of the THF-binding loop specifically associated with the presence of second glutamate moiety (Glu_2_) of the ligand. As shown in **Figure 4**, the loop closes in more tightly against the bound diGlu-FTHF. This is apparent from changes in the position of the backbone of 1-2 Å (**Figure 4A**) as well as in the sidechain of Leu383, a residue in the middle of the loop, which undergoes a significant rotation with a resulting change of ~5 Å in the position of the terminal carbon atoms. In the diGlu-FTHF complex (**Figure 4B**), the Leu383 sidechain (chain A) packs against the ring of Tyr68 (chain B), making a potential CH – π hydrogen bond (**Supplementary Figure 1**) (Brandl et al., 2001). Another hydrogen bond is found between the sidechain of Ser381 and a backbone oxygen of Glu_2_. In addition, two hydrogen bonds are formed between the backbone amides of residues 382 and 383 and the γ-carbonyl of the first glutamate moiety (Glu_1_). A water-mediated contact is found between a carboxyl oxygen of Glu_1_ and the sidechain hydroxyl group of Tyr68.

A detailed comparison of enzyme-ligands contacts in the SHMT8 ternary complexes with FTHF (PDB ID: 6UXJ) and diGlu-FTHF (PDB ID: 8TQF) (**Table 2**) shows that contacts to the pteridine ring of the THF are very similar, while differences emerge in the contacts to the PABA and Glu_1_ moieties. For example, a direct contact between the N atom of PABA and Tyr68 in the FTHF complex is replaced by a water-mediated contact between Tyr68 and an oxygen of Glu_1_ in the diGlu-FTHF complex. Other changes are apparent (**Table 2**; **Supplementary Figure 2**), with the majority being new contacts between the enzyme and diGlu-FTHF, as described above. We note that while Glu_1_ is present in the SHMT8 complex with FTHF, the corresponding contacts are not found, showing that the presence of Glu_2_ is responsible for the novel contacts (e.g., to OEB and O12 atom on **Table 2**).

**Table 2:** SHMT8 hydrogen bond contacts in the ternary complexes with FTHF and diGlu-FTHF.

Ligand atom	Protein residue & atom	Protein Chain	FTHF/A* (Å)	FTHF/B* (Å)	DIGLU/A* (Å)	DIGLU/B* (Å)
Pteridine ring contacts						
N1	N374 ND2	A	3.11	3.11	3.23	3.17
NA2	L129 O	A	2.97	2.90	2.89	2.90
	G133 O	A	3.09	3.04	2.97	3.16
N3	G133 O	A	2.53	2.68	2.77	2.65
O4	L135 N	A	2.90	3.11	2.97	2.81
O5B	E61 OE1	B	2.87	–	2.62	–
	E61 OE2	B	2.56	–	2.85	–
	Y69 OH	B	3.28	–	3.28	–
	H134 NE2	A	2.90	–	2.86	–
N8	N374 OD1	A	2.81	2.83	2.66	2.76
PABA/glutamate contacts						
O	Y139 OH	A	2.56	2.71	2.92	2.94
N/NG1	Y68 OH	B	2.69	2.73	–	–
O11	Water		–	–	2.69	2.69
	(Y68 OH)					
O2/O21	K145 NZ	A	–	–	2.98	2.89
	Water (K145 NZ)		2.62	2.63	–	–
OEB	A382 N	A	–	–	2.90	2.95
	L383 N	A	–	–	2.89	2.86
O12	S381	A	–	–	2.76	2.82

Hydrogen bonds determined by Contact (CCP4) (Winn et al., 2011) for the ligand bound primarily to chain A of PDB IDs: 6UXJ and 8TQF. Additional contacts made by residues in chain B of the obligate dimer are also listed. DIGLU, diGlu-FTHF. *Both ligands were modeled as two conformers (A and B) of approximately equal occupancy due to evidence for two positions of the O5 formyl group that required small shifts in multiple atoms of the pteridine ring. Ligand atom names separated by slash (/) indicates alternate designation for same atom in the two different ligands. Dash indicates missing contact. Water-mediated contacts to atoms in the PABA/glutamate moieties that are conserved in all four active sites are included in the list (participating protein residue in parentheses below). See **Supplementary Figure 2** for schematics with ligand atom names.

### Polyglutamylation of THF increases enzyme stability and folate binding affinity

**Figure 5:**
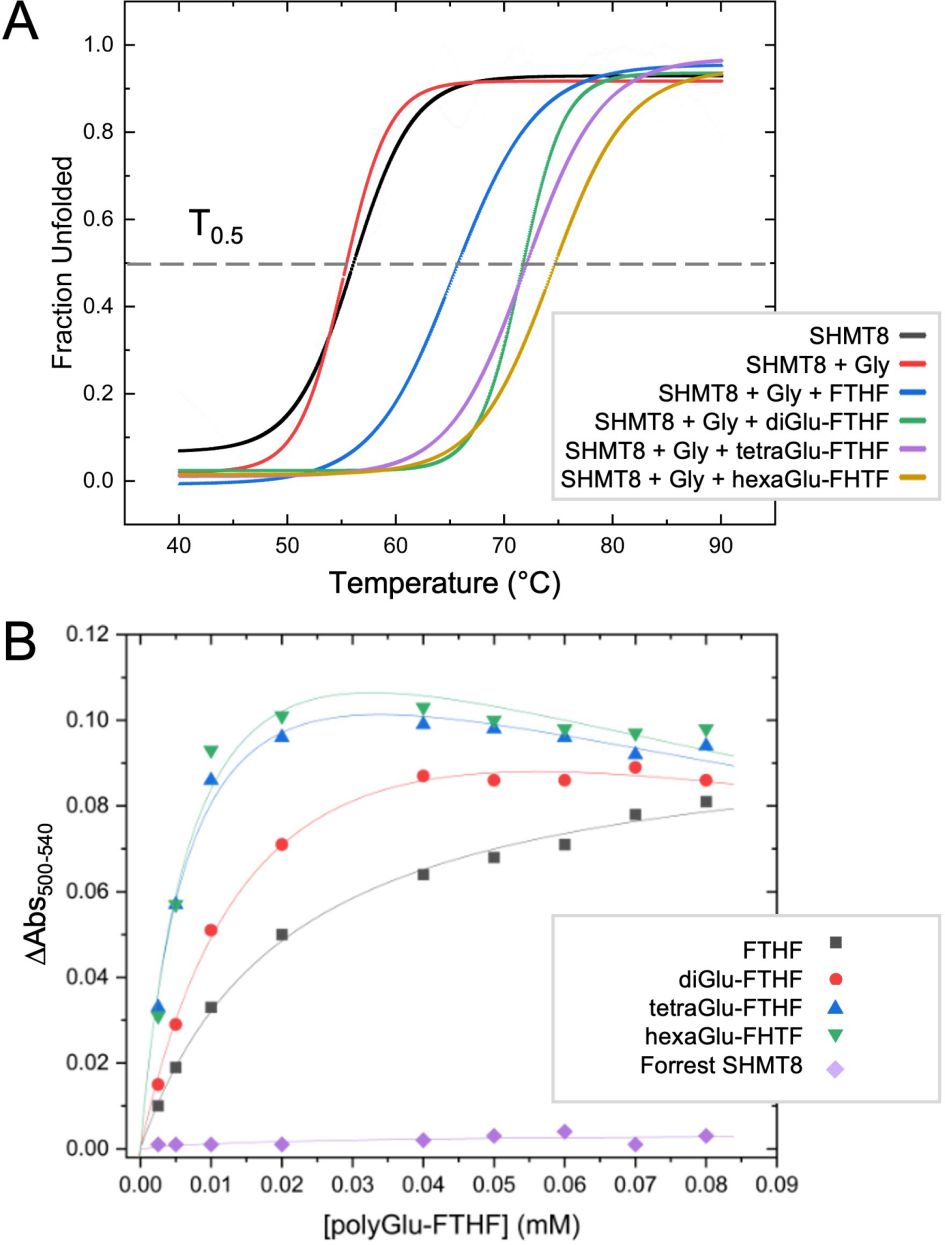
Thermal shift and folate binding data for soybean SHMT with polyglutamylated THF. **(A)** Heat induced melting profiles of SHMT8 in the presence of various ligands. Curves displayed are calculated from the average of three measurements. See panel for key. T_0.5_ (gray line) corresponds to 50% unfolded protein. **(B)** Change in absorbance monitoring binding of various polyGlu-FTHF species. All assays done with SHMT8 from cv. Essex except for the purple line, which was Forrest SHMT8 as noted on key.

To provide further insight into SHMT interactions with polyGlu-THF, we investigated the potential effect of various ligands on the thermal stability of SHMT8. In addition to Gly and FTHF, three polyGlu derivatives of FTHF were tested: di-, tetra-, and hexaGlu-FTHF. T_0.5_ values were determined using a thermal shift assay (TSA) (**Table 3**, **Figure 5A**). SHMT8 and SHMT8 + Gly showed similar T_0.5_. In contrast, addition of FTHF ligands produces significant increases in thermal stability, with changes in T_0.5_ ranging from 9.2 to 18.3°C. The greatest increase in enzyme stability was found for binding of hexaGlu-FTHF, while the smallest was for FTHF. This trend is consistent with previous studies of mammalian SHMT enzymes with various polyGlu-FTHF ligands that show a trend of increased binding affinity with increasing length of the polyglutamate modification (Matthews et al., 1982; Strong and Schirch, 1989; Huang et al., 1998; Fu et al., 2003).

**Table 3:** Melting temperatures (T_0.5_) and dissociation constants for binding of various forms of polyGlu-FTHF to soybean SHMT8.

	FTHF species	T_0.5_ (°C)	Δ T_0.5_	*K*_d_ (μM)
**SHMT8**	–	56.1 ± 0.1	–	–
**SHMT8 + Gly**	–	55.2 ± 0.1	-0.9	–
**SHMT8 + Gly**	FTHF	65.3 ± 0.0	9.2	21 ± 2*
**SHMT8 + Gly**	diGlu-FTHF	71.6 ± 0.1	15.5	20 ± 3
**SHMT8 + Gly**	tetraGlu-FTHF	72.0 ± 0.1	15.9	7 ± 1
**SHMT8 + Gly**	hexaGlu-FTHF	74.4 ± 0.1	18.3	8 ± 2

*K_d_ for FTHF was determined by fitting to the Michaelis-Menten equation. Due to apparent non- Michaelis-Menten behavior, dissociation constants for the polyGlu-FTHF species were fit to the substrate inhibition equation.

Binding affinities for soybean SHMT8 were also determined for di-, tetra-, and hexaGlu-FTHF using a classical spectroscopic assay. This assay monitors the formation of an absorbance peak at 500 nm resulting from formation of a quinonoid species of PLP that accumulates in the presence of bound FTHF (Florini and Vestling, 1957). Data were collected at a single concentration of glycine and dissociation constants (*K*_d_) determined for the various FTHF compounds (**Table 3**, **Figure 5B**). Data for FTHF binding were fit to the Michaelis-Menten equation. Due to apparent non-Michaelis-Menten behavior (**Figure 5B**) of unknown origin, curves for the polyGlu-FTHF species were fit to the substrate inhibition equation. These data show an approximately 3-fold increase in binding affinity to SHMT8 for tetra- and hexaGlu-FTHF over FTHF. In this assay, the *K*_d_ for FTHF and diGlu-FTHF is similar, but due to the different models for fitting, these values may not be directly comparable. Note that the slopes on **Figure 5B** suggest higher affinity binding of diGlu-FTHF than FTHF.

We also conducted binding studies with a double amino variant of soybean SHMT8 found in the soybean cultivar known as Forrest (Liu et al., 2017). These two amino acid polymorphisms (P130R/N358Y) in Forrest SHMT8 are associated with resistance to the soybean cyst nematode (Liu et al., 2012) and previous work has shown that Forrest SHMT8 is highly impaired in binding folate (Korasick et al., 2020). To investigate whether the higher binding affinity of polyGlu-THF might overcome the THF-binding defect of Forrest SHMT8, we assessed FTHF binding using the spectroscopic assay described above. However, no absorbance at 500 nm could be measured (**Figure 5B**, purple line), showing that the defect in THF-binding of Forrest SHMT8 extends to the natural, polyglutamylated form of the substrate found in plant cells.

## Discussion

Polyglutamylation of THF is key to its retention in cells and its localization to various sub-cellular compartments (Green and Matthews, 2007; Mehrshahi et al., 2010). In addition to this essential biological role, polyglutamylation of THF affects interactions with enzymes in the folate cycle. The association of polyGlu-THF with various folate-dependent enzymes has been proposed to increase the stability of the cellular folate pool, since bound folate is less susceptible to oxidative degradation (Orsomando et al., 2005; Mehrshahi et al., 2010). Previous studies suggest that polyglutamylation of THF also affects the kinetic properties of various enzymes in folate metabolism and may control the flux of one-carbon units towards different metabolic pathways (Schirch and Strong, 1989). This modification is therefore an important regulator of THF metabolism and homeostasis in plants and other organisms.

Our previously characterized SHMT8 structures enable detailed comparisons with the diGlu-FTHF complex determined in this study, allowing elucidation of changes associated specifically with the polyglutamate modification. These include the formation of specific enzyme contacts to the diglutamyl tail of FTHF, as well as identification of a novel conformer of the functionally critical THF-binding loop. This conformer, including the large rearrangement of the Leu383 side chain, has not been observed in other crystal structures of SHMT8 or in other SHMT ligand complexes with FTHF. The unique conformer of the THF-binding loop associated with binding of the diglutamylated ligand further highlights the importance of this flexible loop in SHMT function. The novel interactions between diGlu-FTHF and SHMT8 are interesting to consider in the context of the large SHMT enzyme superfamily. Most residues in the active site of SHMT, including those surrounding the PLP and pteridine ring of the folate, are highly conserved (**Supplementary Figure 3**) (Scarsdale et al., 2000; Korasick et al., 2020). On the other hand, residues in the THF-binding loop that interact with the diglutamyl tail of FTHF identified here have generally low to moderate conservation. This is perhaps not surprising as most of the contacts to the glutamates are made by backbone atoms of the protein and are therefore not sequence specific. We note, however, that Tyr68 – the residue involved in contacts with the Leu383 sidechain - is the first residue in a highly conserved YYGG sequence motif in the SHMT family (**Supplementary Figure 3**). The importance of this tyrosine in the activity of human SHMT2 (corresponding residue Tyr105) has been shown through mutagenesis (Katinas et al., 2024).

Prior to this study, the structural details of interactions between polyglutamylated THF and SHMT were largely uncharacterized. The 2.7 Å crystal structure of rabbit SHMT bound to triGlu-FTHF has been reported (Fu et al., 2003), however, electron density for the triglutamyl tail of the ligand is weak (**Supplementary Figure 4**), and there are other indicators of questionable quality in this structure. Thus, is it difficult to draw firm conclusions about interactions with triGlu-FTHF in this complex. In more recent work examining antifolate inhibitors of human SHMT2, crystal structures of two polyGlu derivatives were determined at resolutions of 2.80 and 2.72 Å (Katinas et al., 2024). These structures lack enzyme contacts to this region, but as the inhibitor scaffold differs from folate, their binding mode may not fully reflect that of polyGlu-THF. Neither study found a conformeric change of the THF-binding loop as seen in the SHMT8 complex with diGlu-FTHF. In addition, we note that only half the active sites in the other complexes contain bound ligand (Fu et al., 2003; Katinas et al., 2024), compared with the four fully occupied active sites of the SHMT8 complex bound to diGlu-FTHF. Additional work will be needed to determine whether there are genuine differences between the soybean and mammalian SHMT complexes.

**Figure 6:**
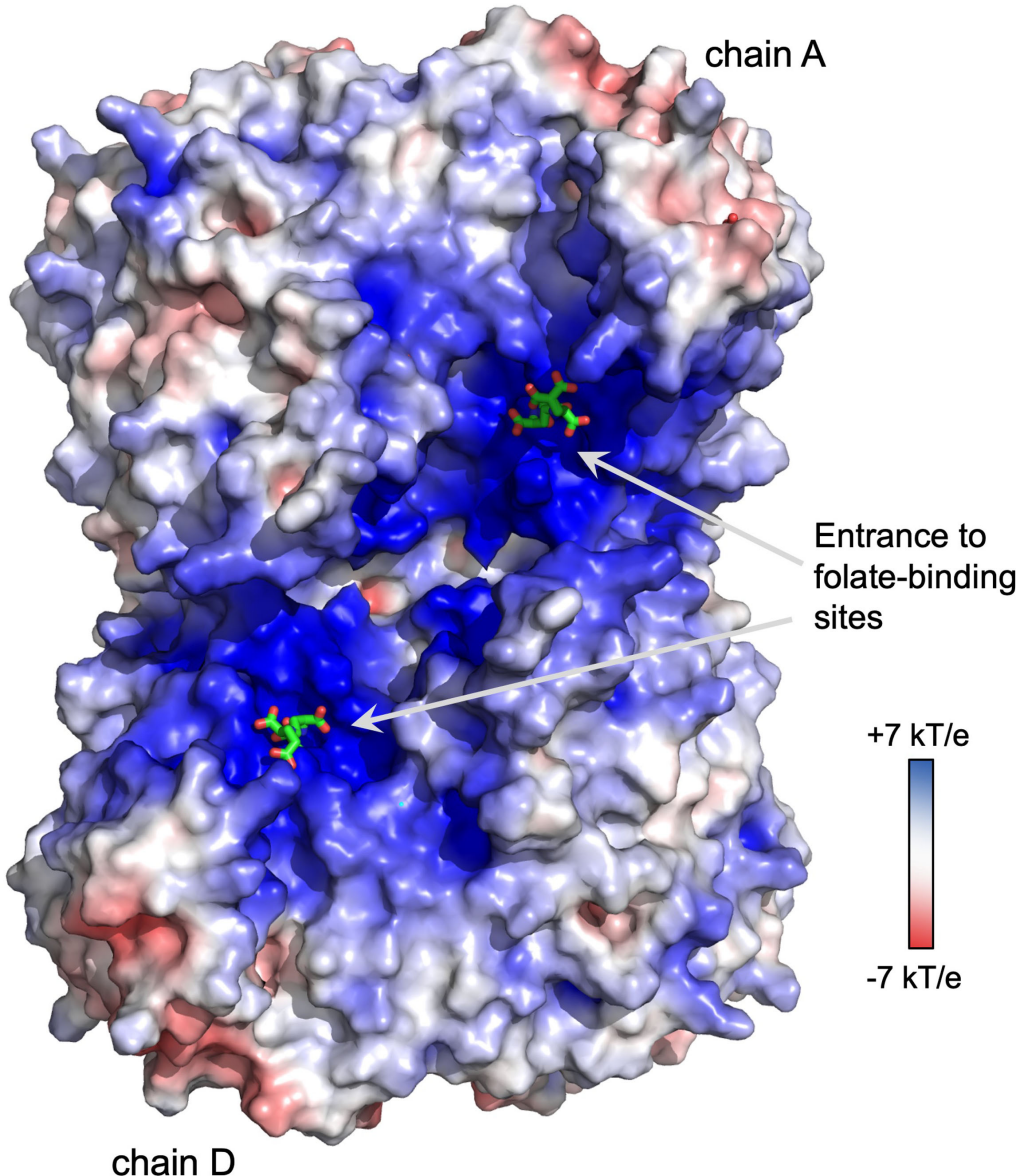
Electrostatic surface potential of the soybean SHMT8 tetramer. Note region of positive potential spanning the two folate binding sites on one side of the tetramer (chains A and D), similar to other eukaryotic SHMTs (Fu et al., 2003; Ruszkowski et al., 2018, 2019). The diGlu-FTHF ligands are shown in green.

The biochemical data presented here complements knowledge gained from various crystal structures of soybean SHMT8. Melting temperatures from TSA show that the stability of the SHMT8-ligand complexes increases substantially in the presence of FTHF relative to ligand-free or PLP-Gly bound enzyme. This is consistent with a variety of small structural changes, including ordering of the THF-binding loop, that occur upon FTHF binding, as noted in the previously determined SHMT8 ternary complex (Korasick et al., 2020). Binding of diGlu-FTHF results in additional stabilization, presumably reflecting the further closure of the THF-binding loop and additional enzyme-ligand contacts. Longer polyGlu tails of the ligand indicate only minor stabilization by TSA but do show increased binding affinity in the FTHF binding assay. Although not visualized in our crystal structure with diGlu-FTHF, the ligand is positioned such that an extended polyGlu tail would be near a region of positive electrostatic charge that runs across the surface of the tetramer, roughly spanning the entrances of the THF-binding sites in chains A and D (**Figure 6**). This feature is highly conserved in the enzyme superfamily and has been previously suggested to interact with polyGlu-THF (Fu et al., 2003; Ruszkowski et al., 2018, 2019). A better understanding of the interactions between SHMT and polyglutamylated THF is highly relevant for inhibitor design. In general, SHMT is a target for antimicrobial (Batool et al., 2020; Jin et al., 2023), antimalarial (Witschel et al., 2015; Schwertz et al., 2017, 2018; Maenpuen et al., 2023) and herbicide agents (Witschel et al., 2015; Li et al., 2024). Human SHMT has also been recognized as a promising target for development of new chemotherapeutics due to the high demand for one-carbon units in cancer cells (Cuthbertson et al., 2021; Zarou et al., 2021; Makino et al., 2022; Situ et al., 2023). Our studies suggest that compounds whose binding permits the conformational change of the THF-binding loop observed in the SHMT8 complex might enable higher affinity binding. Given appropriate closure/rearrangement of the loop, inhibitors modified by polyglutamylation could potentially take advantage of the additional enzyme-ligand interactions as well as the positive electrostatic surface patch of the enzyme. Identifying ligand scaffolds compatible with the loop closure would be a new consideration in the design of tight-binding inhibitors to SHMT. The high-resolution soybean SHMT8 complex with diGlu-FTHF provides a high-quality structural template for future molecular modelling and inhibitor design efforts.

## Data Availability

The datasets presented in this study can be found in online repositories. The names of the repository/repositories and accession number(s) can be found below: https://doi.org/10.2210/pdb8TQF/pdb.
