# Supplementary material for: Structural insights into binding of polyglutamylated tetrahydrofolate by serine hydroxymethyltransferase 8 from soybean
Source: Front Plant Sci. 2024 Aug 19;15:1451839. doi: 10.3389/fpls.2024.1451839 (PMC11366715; doi:10.3389/fpls.2024.1451839)
Supplement: Supplementary file 1 [file Datasheet1.pdf]

Supplementary Material

Structural insights into binding of polyglutamylated tetrahydrofolate by serine hydroxymethyltransferase 8 from soybean

Luckio F. Owuocha, Melissa G. Mitchum, and Lesa J. Beamer*

*** Correspondence:** Lesa J Beamer, beamerl@missouri.edu

Supplementary file 1. Video highlighting conformational change of the THF-binding loop of soybean SHMT. Protein is gray; diGlu-FTHF is magenta. Leu383 is in center of screen. Note how conformational change of Leu383 positions it near ring of the nearby tyrosine (Tyr68). Made in ChimeraX (Meng et al., 2023) using morph command with PDB IDs: 6UXJ and 8TQF.

Supplementary references:

Adams, P. D., Afonine, P. V., Bunkóczi, G., Chen, V. B., Davis, I. W., Echols, N., et al. (2010). PHENIX: A comprehensive Python-based system for macromolecular structure solution. *Acta Crystallographica Section D: Biological Crystallography* 66, 213–221. doi: 10.1107/S0907444909052925

Ben Chorin, A., Masrati, G., Kessel, A., Narunsky, A., Sprinzak, J., Lahav, S., et al. (2020). ConSurf-DB: An accessible repository for the evolutionary conservation patterns of the majority of PDB proteins. *Protein Science* 29, 258–267. doi: 10.1002/pro.3779

Brandl, M., Weiss, M. S., Jabs, A., Sühnel, J., and Hilgenfeld, R. (2001). C-h···π-interactions in proteins. *Journal of Molecular Biology* 307, 357–377. doi: 10.1006/jmbi.2000.4473

Emsley, P., and Cowtan, K. (2004). Coot: Model-building tools for molecular graphics. *Acta Crystallographica Section D: Biological Crystallography* 60, 2126–2132. doi: 10.1107/S0907444904019158

Liebschner, D., Afonine, P. V., Moriarty, N. W., Poon, B. K., Sobolev, O. V., Terwilliger, T. C., et al. (2017). Polder maps: Improving OMIT maps by excluding bulk solvent. *Acta Crystallographica Section D: Structural Biology*. doi: 10.1107/S2059798316018210

Meng, E. C., Goddard, T. D., Pettersen, E. F., Couch, G. S., Pearson, Z. J., Morris, J. H., et al. (2023). UCSF ChimeraX : Tools for structure building and analysis. *Protein Science* 32, e4792. doi: 10.1002/pro.4792

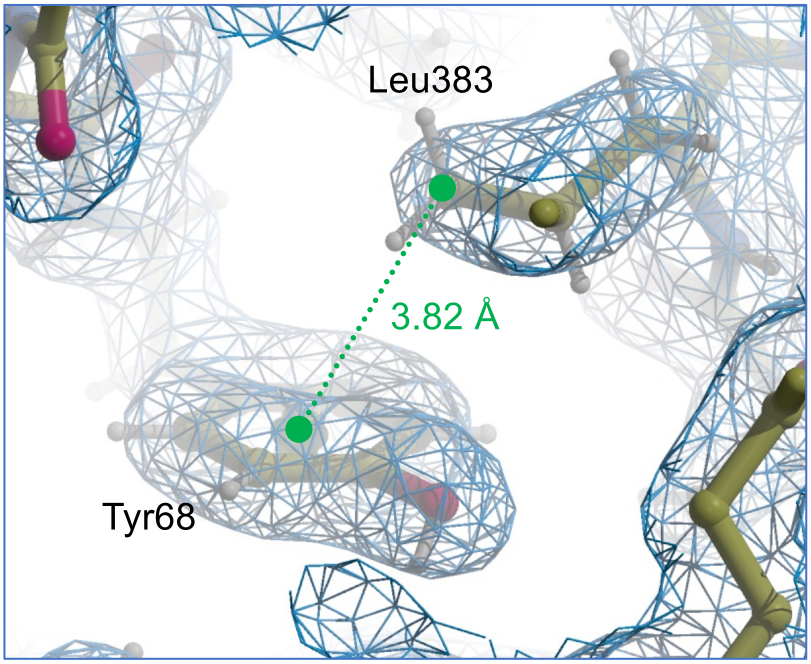

Supplementary Figure 1. A close-up view of the 2Fo-Fc electron density map in the vicinity of the Leu383-Tyr68 interaction in the diGlu-FTHF complex with soybean SHMT8 (PDB ID: 8TQF, chain A). Map is contoured at 1 σ. Calculated hydrogen positions are shown on the model. One of the methyl hydrogens of Leu383 may form a CH-π hydrogen bond with the Tyr68 (e.g., distance from methyl carbon to a point in center of aromatic ring is < 4.5 Å), according to criteria in (Brandl et al., 2001). Figure was made in Coot (Emsley and Cowtan, 2004) using Raster3D option.

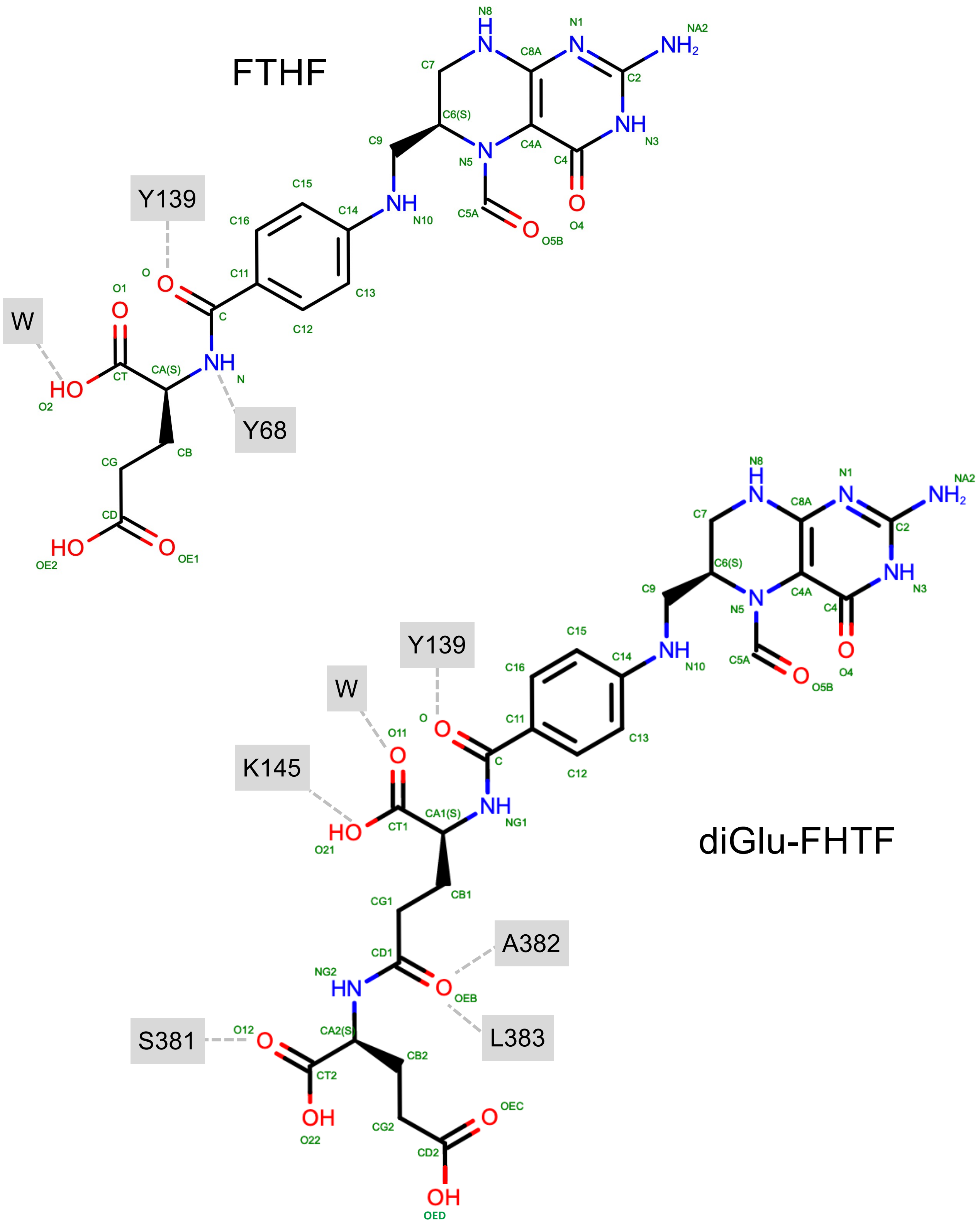

Supplementary Figure 2. Schematic showing molecular structures of FTHF and diGlu-FTHF with corresponding atom names notated. Protein and solvent interactions with the PABA and glutamate moieties are indicated (PDB IDs: 6UXJ and 8TQF) for contacts with FTHF and diGlu-FTHF, respectively. See also Table 3.

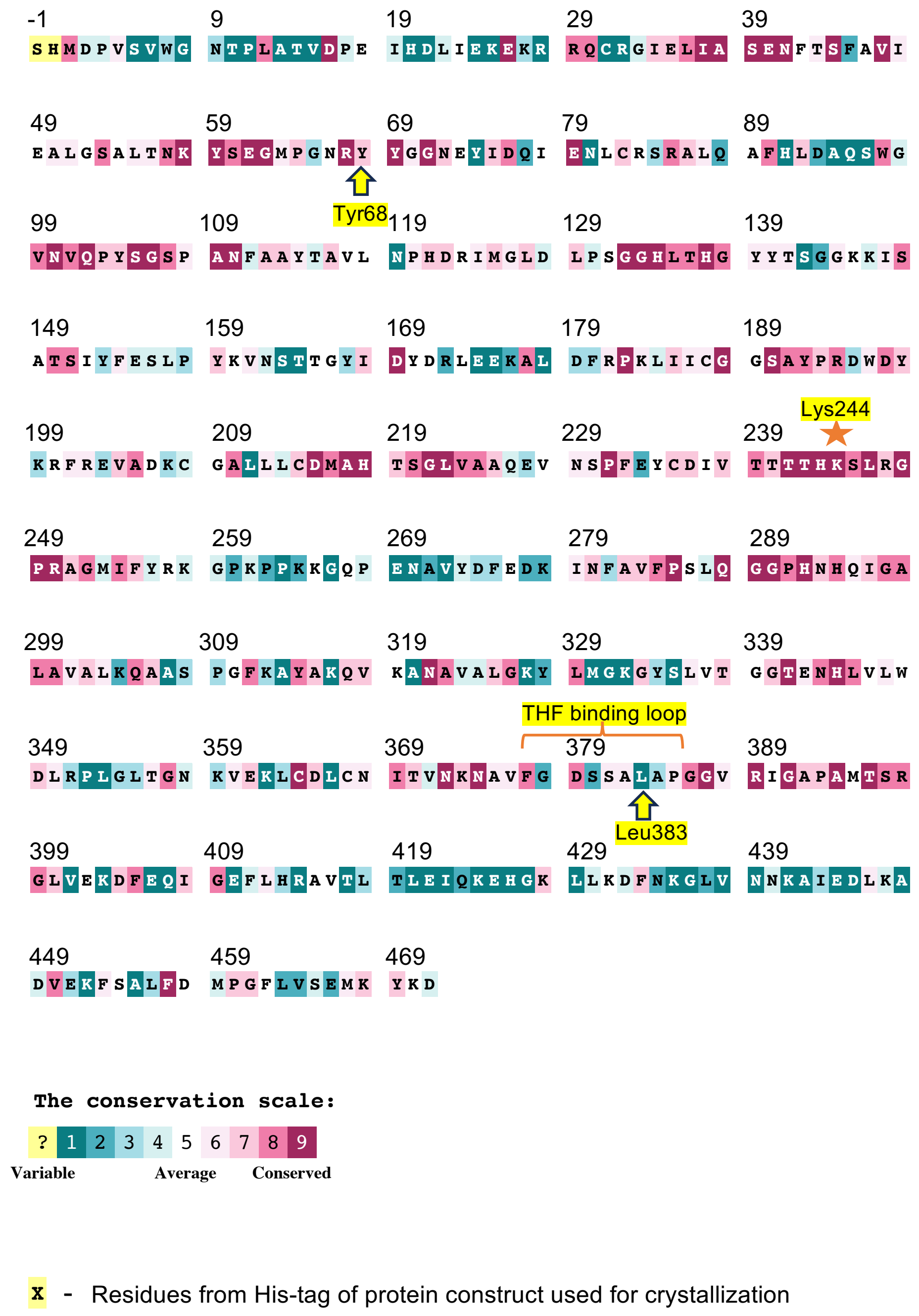

Supplementary Figure 3. Amino acid sequence of soybean SHMT8 colored according to sequence conservation in the SHMT superfamily. Figure made with CONSURF (Ben Chorin et al., 2020) using PDB ID: 6UXJ as input. Key residues/regions are highlighted as follows: Tyr68 and Leu383 (yellow arrows); active site Lys244 (orange star); and the THF-binding loop (orange bracket).

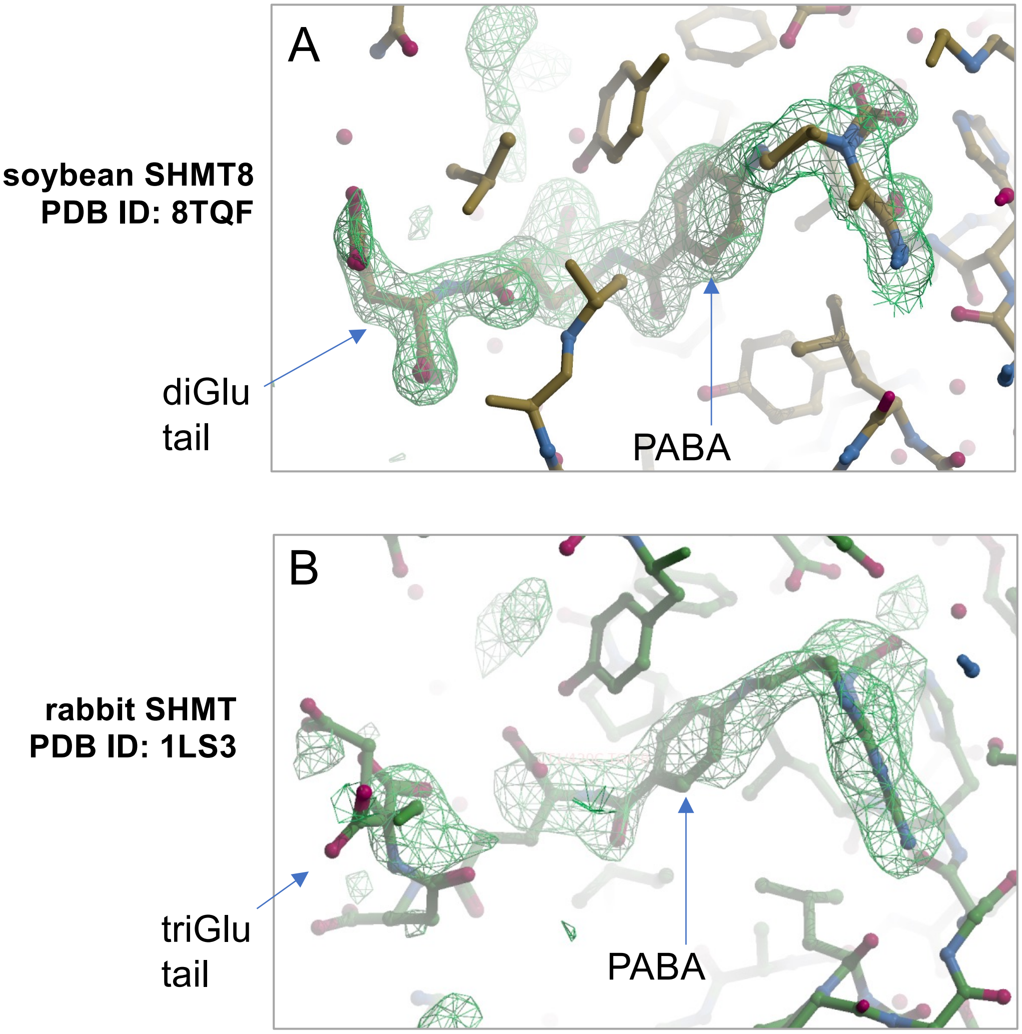

Supplementary Figure 4. Polder style electron maps (Liebschner et al., 2017) of diGlu- and triGlu-FTHF ligands, respectively, in (A) soybean SHMT8 (PDB ID: 8TQF, chain A) and (B) rabbit SHMT (PDB ID: 1LS3, chain B) contoured at 3.0 σ. For reference, the PABA ring of FTHF is highlighted along with the positions of the polyGlu tails. The rabbit SHMT complex lacks continuous density for the triglutamyl moiety and the ligand has multiple with atoms with B-factors >100 Å^2. Polder maps were calculated in PHENIX (Adams et al., 2010); figure was made in Coot (Emsley and Cowtan, 2004) using Raster3D option.
